# Current Clinical Research Directions on Temporomandibular Joint Intra-Articular Injections: A Mapping Review

**DOI:** 10.3390/jcm12144655

**Published:** 2023-07-13

**Authors:** Maciej Chęciński, Kamila Chęcińska, Natalia Turosz, Anita Brzozowska, Dariusz Chlubek, Maciej Sikora

**Affiliations:** 1 Department of Oral Surgery, Preventive Medicine Center, Komorowskiego 12, 30-106 Cracow, Poland; maciej@checinscy.pl; 2 Department of Glass Technology and Amorphous Coatings, Faculty of Materials Science and Ceramics, AGH University of Science and Technology, Mickiewicza 30, 30-059 Cracow, Poland; checinska@agh.edu.pl; 3 Institute of Public Health, Jagiellonian University Medical College, Skawińska 8, 31-066 Cracow, Poland; natalia.turosz@gmail.com; 4 Preventive Medicine Center, Komorowskiego 12, 30-106 Kraków, Poland; brzanita@gmail.com; 5 Department of Biochemistry and Medical Chemistry, Pomeranian Medical University, Powstańców Wielkopolskich 72, 70-111 Szczecin, Poland; sikora-maciej@wp.pl; 6 Department of Maxillofacial Surgery, Hospital of the Ministry of Interior, Wojska Polskiego 51, 25-375 Kielce, Poland

**Keywords:** temporomandibular joint, temporomandibular disorders, intra-articular injections, viscosupplementation, blood preparations, mesenchymal stem cells

## Abstract

This mapping review aims to identify and discuss current research directions on intracavitary temporomandibular joints (TMJs) injections. The inclusion criteria allowed studies published in the last full six years, based on patients diagnosed with temporomandibular joint disorders (TMDs), treated by TMJ intra-articular injections. Medical databases covered by the Association for Computing Machinery, Bielefeld Academic Search Engine, PubMed, and Elsevier Scopus engines were searched. The results were visualized with tables, charts, and diagrams. Of the 2712 records identified following the selection process, 152 reports were qualified for review. From January 2017, viscosupplementation with hyaluronic acid (HA) was the best-documented injectable administered into TMJ cavities. However, a significant growing trend was observed in the number of primary studies on centrifuged blood preparations administrations that surpassed the previously leading HA from 2021.

## 1. Introduction

### 1.1. Background

The temporomandibular joints (TMJs) connect the mandible to the temporal bones. These joints are essential to the proper functioning of the stomatognathic system, including opening and closing the mouth, chewing, and speaking [1]. Rotation and slide in TMJs are palpable on both sides in the preauricular area during abduction and adduction of the mandible [2,3]. Each TMJ consists of the mandibular condyle, the articular fossa of the temporal bone, and the cartilage disc that separates the two bones and cushions them during movement [1,4]. The joint is surrounded by a network of muscles, ligaments, and nerves that help stabilize and control its function [1,5].

Temporomandibular disorders (TMDs) are a collective term for a group of conditions manifested by abnormal function of the temporomandibular joints (TMJs) [6,7]. According to the meta-analysis by Valesan et al., the overall prevalence of TMDs in the adult population is approximately 31% [6] The causes of TMDs are seen primarily in malocclusions, morphological abnormalities, and post-traumatic changes within TMJs, and masticatory muscle dysfunction [5,8,9,10]. The causes of TMDs should also be sought in general deterioration of health (including psychological burden) and limited access to medical care, which could be observed with an increase in the frequency of TMDs diagnoses during the COVID-19 pandemic, according to the study by Haddad et al., to about 42% [8,9]. However, Ginszt et al. showed that there is a certain mechanical effect of wearing medical masks on muscle activity, in particular the anterior part of the temporalis muscle, which may also be important for the increase in the incidence of TMDs [10]. TMDs can manifest as articular and/or muscular pain, acoustic symptoms from TMJs, and reduced chewing quality [5,11,12]. Amongst TMDs treatment methods are biofeedback, cognitive–behavioral therapy, physiotherapy, oral drug therapy, splint therapy, changing the occlusive conditions, and minimally invasive, arthroscopic, or open surgery [13,14,15,16,17].

Minimally invasive intra-articular manipulations are currently considered a viable alternative in the management of TMDs, especially when more conservative treatments fail to provide relief from TMDs symptoms [18,19,20]. These techniques include arthrocentesis and intra-articular injections [20,21]. Arthrocentesis consists in rinsing the joint cavity with infusion fluids using two- and one-needle methods [20,21]. Intra-articular injections involve injecting the drug directly into the TMJ cavity [22,23]. Intra-articular injections are indicated to relieve joint pain, suppress inflammation and improve joint function [22,23].

Various substances are administered intra-articularly, including corticosteroids (CSs), hyaluronic acid (HA), and blood products such as platelet-rich plasma (PRP) or injectable platelet-rich fibrin (I-PRF) [17,18,24]. CSs are known for their strong anti-inflammatory effect. Supplementation of the main component of synovial fluid, HA, improves the mobility of joint surfaces relative to each other [24,25]. PRP and I-PRF, differing in composition resulting from the preparation, have the added advantage of being highly safe due to their autogenous nature [17,24,25].

### 1.2. Rationale

Scientific articles published in recent years indicate a sudden increase in the number of substances administered intra-articularly, and surgical technique is constantly improving. The growing number of primary research papers demonstrates the increasing popularity of intra-articular injections. Therefore, it seems reasonable to frequently update the state of knowledge about injection techniques in the treatment of TMDs. To the knowledge of the authors of this paper, no systematic map on this subject has been published to date.

### 1.3. Objectives

This mapping review aims to identify and discuss current research directions on intracavitary TMJs injections.

## 2. Materials and Methods

The systematic map was prepared by: (1) defining eligibility criteria; (2) developing a search strategy; (3) searching medical databases using leading engines; (4) selecting reports according to predetermined criteria; (5) assessing the research level of evidence; (6) synthesizing the results; (7) presenting the main research directions.

### 2.1. Eligibility Criteria

The eligibility criteria were established in accordance with the PICOS methodology (Table 1) [26,27,28]. Studies based on patients diagnosed with TMDs were included. Due to the different etiology and treatment, patients with TMDs as a manifestation of a general disease, e.g., rheumatoid arthritis or juvenile idiopathic arthritis, were excluded. Cadaver, animal, or in vitro studies were omitted as not including patients. Systematic reviews and meta-analyses based on eligible studies were included. Interventions containing the administration or administrations into the temporomandibular joint cavity were included. Additional interventions of a different kind were allowed, such as physiotherapy, pharmacotherapy, splint therapy, etc. Arthrocentesis alone, without intra-articular administration of any substance, was excluded. More invasive intra-articular manipulations, i.e., arthroscopy or open surgery, were disqualified. Due to the inclusion of studies with varying levels of evidence, the criterion of comparison was not applicable. Changes in any TMDs severity index were allowed as an outcome. Single case reports and any series less than 4 cases were rejected. In order to demonstrate the current directions of research, reports published in the last full 6 years, i.e., from 1 January 2017, to final searches conducted on 13 March 2023, were included.

### 2.2. Search Strategy

The search strategy was based on terms identifying TMJ and injections. In its basic form, the query was:

“(temporomandibular OR TMJ OR TMJs) AND (injection OR injections OR puncture OR punctures OR administration OR administrations)”.

The following search engines were used: (1) Association for Computing Machinery: Guide to Computing Literature (ACM; 3,470,491 records) [29]; (2) Bielefeld Academic Search Engine (BASE; 320,685,924 records) [30]; (3) National Library of Medicine: PubMed (NLM; over 35,000,000 records) [31]; (4) Elsevier Scopus (ES; over 87,000,000 records) [32,33]. For each search engine, the necessary query modifications were made to ensure the validity of the search (Table A1). Filters were used to exclude studies published before 2017, where possible.

### 2.3. Selection Process

Reports were selected for the systematic map in two stages by two authors (M.C. and A.B.) using Rayyan tool [34]. Screening consisted of including abstracts according to PICOS criteria. Acceptance by any of the judges resulted in the promotion of the record to the eligibility stage. In case of discrepancies regarding inclusion, decisions were made by consensus, with the casting vote of the third investigator (K.C.).

### 2.4. Qualification of Reports Due to the Study Design

The information on the design of the studies included in the review was extracted from the source reports by two authors (M.C. and K.C.) and unified using the Oxford Centre for Evidence-Based Medicine 2011 Levels of Evidence scale [35]. Systematic reviews involving randomized controlled trials were qualified as Level 1. Levels 2–4 were assigned to randomized controlled, non-randomized controlled, and uncontrolled trials, respectively.

### 2.5. Syntheses

The results of this mapping review were tabularized and illustrated by an organizational chart representing the research directions forks falling within the eligibility criteria described above. The numbers of individual articles in particular forks were presented with a bar, bubble, and column charts, with trend lines indicating the dominant directions of primary research on the last one.

## 3. Results

**Figure 1:**
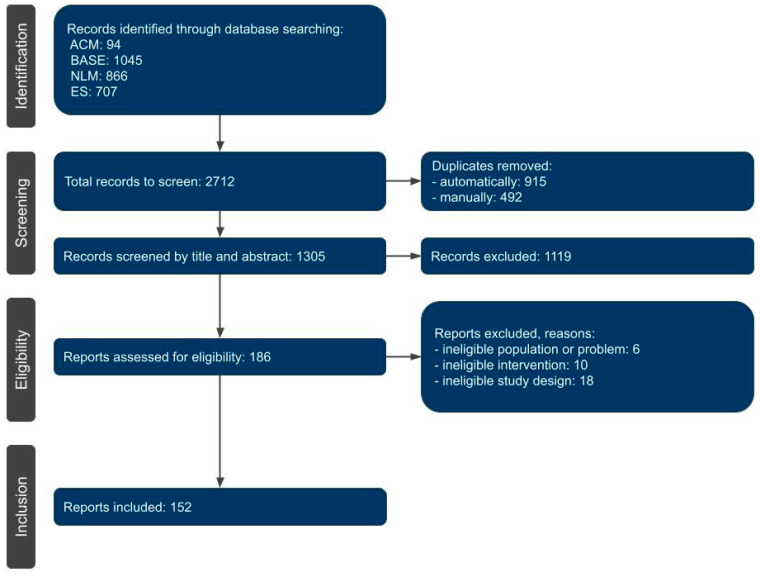
Flow diagram of the selection process. ACM—Association for Computing Machinery: Guide to Computing Literature; BASE—Bielefeld Academic Search Engine; NLM—National Library of Medicine: PubMed; ES—Elsevier Scopus.

**Figure 2:**
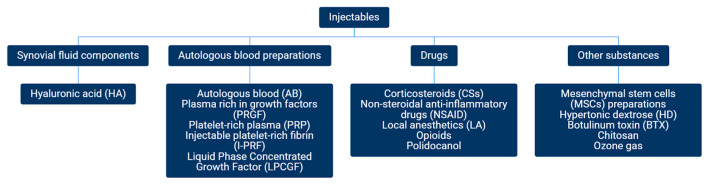
Classification of injectables (based on the included reports).

**Figure 3:**
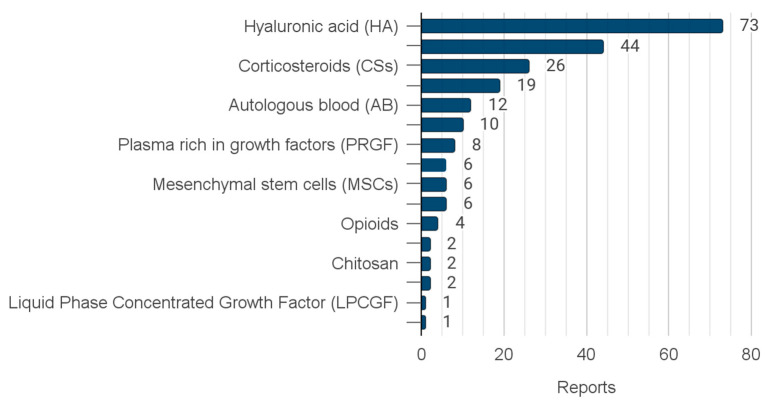
The number of reports on individual injectables.

**Figure 4:**
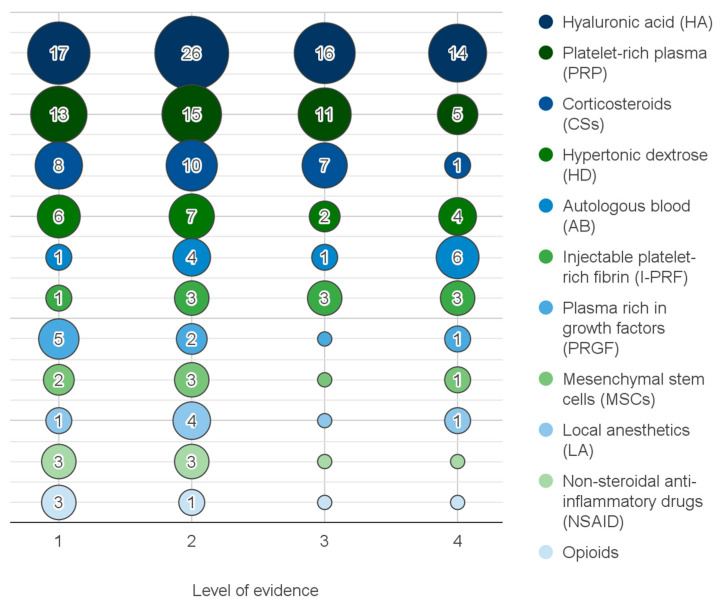
The number of reports by the level of evidence (horizontal axis) and injectables (vertical axis). Reports on injectables evaluated in less than 3 papers not included.

**Figure 5:**
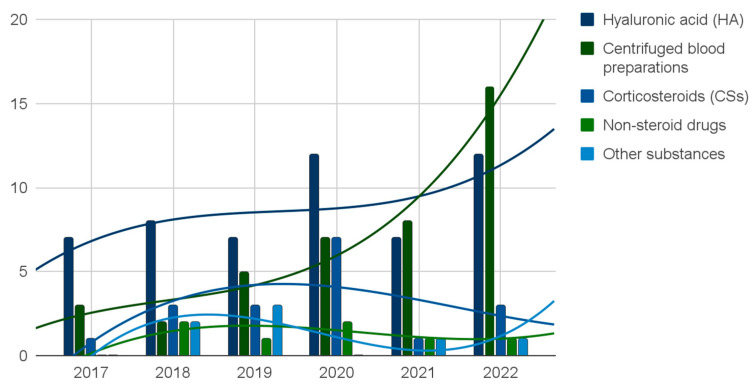
The number of primary research reports (level of evidence 2–4) on individual mandibular hypomobility treatment injectables with third-degree polynomial trendlines.

Of the 2712 records identified, 152 reports were ultimately qualified for the mapping review, with 32, 53, 28, and 39 reports in levels of evidence from 1 to 4, respectively (Figure 1, Figure 2, Figure 3, Figure 4 and Figure 5, Table A2) [18,19,22,23,24,25,36,37,38,39,40,41,42,43,44,45,46,47,48,49,50,51,52,53,54,55,56,57,58,59,60,61,62,63,64,65,66,67,68,69,70,71,72,73,74,75,76,77,78,79,80,81,82,83,84,85,86,87,88,89,90,91,92,93,94,95,96,97,98,99,100,101,102,103,104,105,106,107,108,109,110,111,112,113,114,115,116,117,118,119,120,121,122,123,124,125,126,127,128,129,130,131,132,133,134,135,136,137,138,139,140,141,142,143,144,145,146,147,148,149,150,151,152,153,154,155,156,157,158,159,160,161,162,163,164,165,166,167,168,169,170,171]. In the selection process, a total of 1407 duplicates were rejected, mainly due to overlapping search engines. At the screening stage, 1119 entries not related to TMJs injection treatment were excluded; these were present due to the intentionally unrestrictive choice of keywords in the queries. At the very end of the selection, 34 articles (mainly case reports) were rejected in the course of the full-text evaluation in accordance with the adopted inclusion and exclusion criteria.

## 4. Discussion

TMDs that cause articular pain and mandibular mobility limitation are two main reasons for delivering intra-articular injections, except for HD and AB administrations which are performed to treat recurrent subluxation of the temporomandibular joint [22,24,37,40,44,53,55,57,61,65,66,69,72,73,90,94,96,97,110,111,131,149,160,165,168]. Currently, there is an intensive search for the gold standard of TMDs treatment, which is difficult due to the variety of etiologies and the specificity of individual dysfunctions. The main directions of research on the use of intra-articular injections in this indication are presented below.

### 4.1. Hyaluronic Acid (HA) Viscosupplementation

Improving the composition of the synovial fluid by supplementing its main ingredient, HA, is the most frequently described type of injection into the TMJs. 56 primary studies, including 26 randomized, summarized in 17 systematic reviews make this injectable substance the best studied. There is a steady upward trend in the number of primary studies on HA published in subsequent years. The primacy of HA from 2021 seems to be threatened by centrifuged blood products, but this group of substances is heterogeneous and cannot be compared to HA in terms of effectiveness as a whole [18,19,22,25,38,43,49,51,54,58,59,60,63,68,70,71,74,75,78,80,80,82,86,88,89,91,92,93,95,98,99,100,101,102,105,106,110,113,114,115,116,117,120,121,124,126,127,129,130,132,133,134,135,137,138,143,144,146,151,152,153,157,158,161,163,164,166,171].

### 4.2. Hypertonic Dextrose (HD) Prolotherapy

Unlike viscosupplementation, HD prolotherapy aims to reduce the range of motion of the mandible. The administration of HD as an irritant is one of the treatment methods for hypermobility in TMJs. So far, the concentration of HD has not been standardized and varies from 12.5% to 25%. Only the studies involving the administration of HD into the TMJs are included in this review, but the substance is frequently deposited peri-articularly in this indication. Of the two substances applied to TMJs (HD and autologous blood), HD injections are better documented. The 13 primary studies on intra-articular administration of HD have been summarized in 6 reviews [22,24,37,53,65,66,72,73,94,96,97,110,111,131,149,160,165].

### 4.3. Blood Preparations Autotransplantation

Blood preparations are a group of substances obtained from autologous peripheral blood including unprocessed blood and blood concentrates. Autologous blood (AB) is the second, next to HD, substance administered into the TMJs for the treatment of hypermobility. Reports since 2017 describing AB therapy are fewer and generally with a lower level of evidence than these regarding HD (four randomized, one non-randomized, and six uncontrolled) [37,40,44,55,57,61,65,66,69,90]. The only included systematic review on AB therapy administrated intra-articularly suggests the need for randomized trials [168].

Blood concentrates are obtained by centrifuging freshly taken venous blood and are delivered immediately after the preparation. Different protocols allow obtaining various concentrates without the red cell fraction. Some of the concentrates can be collected in liquid form and injected into TMDs. In the discussed years, the administration of preparations referred to as plasma rich in growth factors (PRGF), PRP, I-PRF and liquid phase concentrated growth factor (LPCGF) into the TMJs cavities was described. They differ in the centrifugation procedure, and thus in the composition and effectiveness in anti-inflammatory action and stimulation of tissue regeneration. The lack of a standardized centrifugation protocol for platelet-rich concentrates for injection into TMJs clearly illustrates the active development of a therapeutic standard. Of the 152 reports on blood concentrates included, 120 primary studies were published (including 53 randomized trials) as well as 32 systematic reviews. From 2021, primary research on the substances in question has been more numerous than on HA. [18,23,25,36,46,56,60,62,64,67,68,70,74,75,81,84,85,87,95,101,103,104,107,112,113,114,118,119,122,123,123,128,133,134,135,136,137,141,142,143,145,147,148,152,158,161,162,167,169,170].

### 4.4. Mesenchymal Stem Cells (MSCSs) Autotransplantation

MSCs, obtained primarily from autogenous fat, are an attractive injectable due to their high potential to stimulate the regeneration of TMJ structures. Only four primary studies using MSCs for intracavitary administration are known, of which three were randomized.

### 4.5. Drugs Administration

Substances used as drugs for other indications, normally with other routes of administration, are included in this group. CSs are definitely the best studied among them. After HA and PRP, CSs were the third most frequently reported injectables group in 2017–2022 (8 systematic reviews, 10 randomized trials, and 8 other trials) [19,25,38,39,41,45,47,47,49,51,54,62,75,86,121,125,127,134,135,137,140,156,164,166,171]. Nevertheless, since 2020, the number of primary studies on intra-articular injection of CSs has clearly decreased. Other papers describe the use of non-steroidal anti-inflammatory drugs (NSAIDs), local anesthetics (LAs), opioids, and polidocanol. These substances have been used exceptionally and so far there is no well-established knowledge about their effectiveness and safety [24,37,38,62,73,77,79,97,101,108,137,150,165].

### 4.6. Other Substances Injections

Unique studies on the administration of botulinum toxin (BTX), chitosan, and ozone gas provide potential directions for the future development of intra-articular injections. At present, however, these methods should be regarded as insufficiently researched [24,42,83,139,159].

### 4.7. Limitations

This systematic map was limited to injections into the temporomandibular joint cavities. Therefore, studies focusing on pericapsular injections, which are used in the treatment of mandibular hypermobility, were omitted. Therefore, this paper covers only a part of the articles on AB and HD injections.

A separate large group of interventions, not included in this review, is stand-alone arthrocentesis. They have been excluded as there was no intention to administer any substance intra-articularly. However, TMJs lavage relieves pain and increases mandibular mobility similarly to injections of, for example, HA or PRP, and future mapping of papers on this topic should be considered.

## 5. Conclusions

In the years 2017–2023, hyaluronic acid was the most common topic of scientific publications among injectables administered into temporomandibular joint cavities (26 randomized controlled trials and 30 other clinical studies). In the same period, there was a significant upward trend in the number of published primary studies focused on centrifuged blood preparations used in the treatment of TMDs. As of 2021, blood products administered into TMJs cavities have become a more popular topic for professional medical articles than hyaluronan. Nevertheless, it should be emphasized that this is a group of substances that differ in composition depending on the centrifugation protocol. The therapeutic efficacies of substances evaluated in at least three clinical trials were synthesized in systematic reviews.

## Figures and Tables

**Table 1:** Review eligibility criteria.

Domain	Inclusion Criteria	Exclusion Criteria
Population	Patients diagnosed with TMDs	TMDs as a systemic disease component
Intervention	TMJ intra-articular injection	Arthrocentesis alone or more invasive interventions, e.g., arthroscopy
Comparison	Any or none	Not applicable
Outcomes	TMDs severity assessment	Not applicable
Settings	Reports based on 4 or more cases	Reports published before 2017

## Data Availability

All collected data are included in the content of this article.

## Appendix A

**Table A1:** Search queries.

Engine	Inclusion Criteria
ACM	[[All: temporomandibular] OR [All: tmj] OR [All: tmjs]] AND [[All: injection] OR [All: injections] OR [All: puncture] OR [All: punctures] OR [All: administration] OR [All: administrations]] AND [E-Publication Date: (1 January 2017 TO 31 December 2023)]
BASE	(temporomandibular OR TMJ OR TMJs) AND (injection OR injections OR puncture OR punctures OR administration OR administrations) year: [2017 TO *]
NLM	(temporomandibular OR tmj OR tmjs) AND (injection OR injections OR puncture OR punctures OR administration OR administrations) AND (“1 January 2017” [Date—Publication]: “3000” [Date—Publication])
ES	TITLE-ABS-KEY ((temporomandibular OR tmj OR tmjs) AND (injection OR injections OR puncture OR punctures OR administration OR administrations)) AND PUBYEAR > 2016

**Table A2:** Included reports.

First Author	Publication Year	Level of Evidence	Injectables	DOI Number
Bayramoglu	2023	2	NSAID	10.1186/s12903-023-02852-z
Bhargava	2023	2	AB, HD, LA	10.1007/s12663-022-01738-x
Chęciński	2023	1	HA, HD	10.3390/jcm12041664
Chhapane	2023	2	AB, HD	10.1007/s12663-023-01848-0
Gupta	2023	2	NSAID	10.7759/cureus.34580
Hegab	2023	2	HA, PRP	10.1016/j.jormas.2022.11.016
Li	2023	4	HA	10.1007/s11282-022-00621-2
Vingender	2023	3	HA, PRP, I-PRF	10.1016/j.jcms.2023.01.017
Wu	2023	4	Chitosan	10.3390/jcm12041657
Abbadi	2022	2	PRP	10.7759/cureus.31396
Ansar	2022	3	PRP	10.25122/jml-2021-0240
Asadpour	2022	2	HA, PRP	10.1016/j.joms.2022.05.002
Bera	2022	4	I-PRF	10.1111/ors.12665
Mazzara Bou	2022	1	CSs	10.20986/recom.2022.1344/2022
Castaño-Joaqui	2022	4	HA	10.1016/j.jcms.2022.06.004
Cen	2022	3	HA	10.1007/s00784-021-04241-8
Chęciński	2022	1	MSCs	10.3390/cells11172709
Chęciński	2022	1	HD, LA, MSCs, NSAID, ozone, opioids	10.3390/jcm11092305
Chęciński	2022	1	HA	10.3390/jcm11071901
Dasukil	2022	2	HA, PRP	10.1016/j.jcms.2022.10.002
Dharamsi	2022	2	CSs, HA	10.1007/s12663-022-01804-4
Ferreira	2022	3	HA	10.1080/08869634.2022.2141784
Ghoneim	2022	2	I-PRF	10.1016/j.jds.2021.07.027
Gutiérrez	2022	1	PRP, PRGF	10.1016/j.jormas.2021.12.006
Haggag	2022	2	HD	10.1016/j.jcms.2022.02.009
Hyder	2022	2	HA	10.1016/j.jormas.2022.05.007
Işık	2022	2	I-PRF	10.1016/j.jcms.2022.06.006
Jacob	2022	2	HA, PRP	10.1007/s12663-021-01519-y
Shah	2022	4	AB	10.4103/jiaomr.jiaomr_199_21
Leketas	2022	2	HA, PRGF	10.1080/08869634.2022.2081445
Liu	2022	3	PRP	10.1111/joor.13261
Macedo de Sousa	2022	3	CSs, HA, PRP	10.3390/life12111739
Manafikhi	2022	4	I-PRF	10.1186/s12891-022-05421-7
Massé	2022	1	CSs, HA, HD, PRP	N/A
Memiş	2022	4	HA	10.1016/j.jcms.2022.07.003
Memiş	2022	3	HD	10.5125/jkaoms.2022.48.1.33
Pandey	2022	4	AB, HD	10.4103/njms.njms_509_21
Rajput	2022	2	PRP	10.1007/s12663-020-01351-w
Ramakrishnan	2022	2	HA, PRP	10.4103/njms.NJMS_94_20
Sari	2022	3	BTX	10.1016/j.jormas.2022.04.019
Sharma	2022	4	AB	10.1007/s12663-021-01540-1
Sikora	2022	4	PRP	10.3390/ijerph192013299
Sikora	2022	4	PRP	10.3390/jcm11154281
Singh	2022	2	CSs	10.4103/njms.njms_291_21
Vaidyanathan	2022	4	Polidocanol	10.4103/ams.ams_138_22
Xie	2022	1	CSs, HA, PRP	10.1016/j.jebdp.2022.101720
AbdulRazzak	2021	3	CSs	10.1007/s10006-020-00901-3
Al-Hamed	2021	1	PRP, PRGF	10.1177/2380084420927326
Amer	2021	2	AB	10.21608/EJENTAS.2021.56244.1300
Chandra	2021	3	PRP	10.4103/jfmpc.jfmpc_1633_20
Cömert Kılıç	2021	2	HA	10.1016/j.jcms.2021.02.012
Dasukil	2021	4	HD, LA	10.1007/s12663-020-01328-9
Derwich	2021	1	CSs, HA, PRP	10.3390/ijms22147405
Ferreira	2021	4	HA	10.1080/07853890.2021.1897446
Goker	2021	1	HA	10.23812/21-2supp1-3
Harba	2021	3	HA, PRP	10.17219/dmp/127446
Karadayi	2021	2	I-PRF	10.1016/j.jcms.2021.01.018
Li	2021	3	PRP	10.1016/j.joms.2020.09.016
Liapaki	2021	1	HA, PRP	10.1016/j.ijom.2021.01.019
Liu	2021	1	NSAID, opioids	10.1111/joor.13105
Romero-Tapia	2021	3	CSs, HA	10.5005/JP-JOURNALS-10024-2890
Rossini	2021	4	HA	10.6061/clinics/2021/e2840
Sàbado-Bundó	2021	1	HA	10.1080/08869634.2021.1925029
Sarwar	2021	3	PRP	10.51253/pafmj.v71i4.5361
Sembronio	2021	2	MSCs	10.1016/j.joms.2021.01.038
Singh	2021	2	HA	10.4103/jpbs.JPBS_675_20
Singh	2021	2	PRP	10.1007/s12663-019-01320-y
Sit	2021	1	HD	10.1038/s41598-021-94119-2
Torul	2021	3	HA, I-PRF	10.1016/j.ijom.2021.03.004
Wang	2021	4	HA	10.1016/j.bjoms.2020.07.013
Zubair	2021	4	PRP	10.21276/apjhs.2021.8.2.2
Aamir	2020	4	AB	N/A
Abrahamsson	2020	1	AB, HD	10.1007/s00784-019-03126-1
Ahmed	2020	2	PRGF	N/A
Albilia	2020	4	I-PRF	10.1080/08869634.2018.1516183
Bukhari	2020	3	AB	10.5455/JPMA.5002
Dolwick	2020	2	CSs	10.1016/j.joms.2020.02.022
Fayed	2020	2	HA, opioids	N/A
Hammoodi	2020	3	CSs, PRP	N/A
Hosgor	2020	3	HA	10.1016/j.jcms.2020.07.008
Jara Armijos	2020	1	HA, PRP, PRGF, NSAID	10.4321/s0213-12852020000100005
Li	2020	1	PRP	10.11607/ofph.2470
Liu	2020	1	CSs, HA, PRGF, opioids	10.1016/j.joms.2019.10.016
Marzook	2020	3	CSs, HA	10.1016/j.jormas.2019.05.003
Mohammed	2020	2	CSs, HA	10.37506/ijfmt.v14i2.2817
Pihut	2020	2	HA, PRP	10.3390/ijerph17134726
Santagata	2020	4	HA	10.3390/jfmk5010018
Sezavar	2020	2	PRP	10.29252/jrdms.5.3.7
Sikora	2020	4	HA	10.3390/jcm9061749
Singh	2020	3	CSs, HA	10.1007/s12070-019-01738-3
Macedo De Sousa	2020	2	CSs, HA, PRP	10.3390/medicina56030113
Stasko	2020	3	HA	10.4149/BLL_2020_056
Taşkesen	2020	3	HD	10.1080/08869634.2020.1861887
Yuce	2020	3	HA, I-PRF	10.1097/SCS.0000000000006545
Zarate	2020	2	HD, LA	10.1089/acm.2020.0207
Zigmantavičius	2020	1	HA, PRP	N/A
Abd	2019	2	PRP	N/A
Bergstrand	2019	2	HA	10.2334/josnusd.17-0423
Brignardello-Petersen	2019	1	I-PRF	10.1016/j.adaj.2019.01.015
Carboni	2019	2	MSCs	10.1097/SCS.0000000000004884
Chung	2019	1	PRP	10.1016/j.oooo.2018.09.003
De Riu	2019	2	MSCs	10.1016/j.jcms.2018.11.025
Gavin Clavero	2019	4	HA	10.1007/s10006-019-00789-8
Giacomello	2019	4	PRGF	N/A
Gokçe Kutuk	2019	2	CSs, HA, PRP	10.1097/SCS.0000000000005211
Henk	2019	4	CSs	10.5935/0946-5448.20190003
Isacsson	2019	2	CSs	10.1111/joor.12718
Khallaf	2019	4	PRP	N/A
Louw	2019	2	HD, LA	10.1016/j.mayocp.2018.07.023
Mahmmood	2019	4	MSCs	10.1097/SCS.0000000000004938
Sequeira	2019	4	HA	10.1007/s12663-018-1093-4
Su	2019	4	HA	10.11607/ofph.2044
Toameh	2019	2	HA, PRP	10.17219/dmp/109329
Yilmaz	2019	2	HA	10.1016/j.jcms.2019.07.030
Batifol	2018	4	BTX	10.1016/j.jormas.2018.06.002
Bousnaki	2018	1	HA, PRP	10.1016/j.ijom.2017.09.014
Brignardello-Petersen	2018	1	PRP	10.1016/j.adaj.2017.11.012
Cen	2018	2	HA	10.1111/odi.12760
Daif	2018	4	Ozone	10.7203/jo3t.2.2.2018.11132
Davoudi	2018	1	CSs	10.4317/medoral.21925
Ferreira	2018	1	HA	10.1016/j.jcms.2018.08.007
Fonseca	2018	4	HA	10.1155/2018/5392538
Fouda	2018	2	HD	10.1016/j.bjoms.2018.07.022
Ganti	2018	3	HA	10.5005/jp-journals-10024-2456
Gupta	2018	2	CSs, LA, PRP	10.4103/njms.NJMS_69_16
Haigler	2018	1	PRP, PRGF	10.1016/j.adaj.2018.07.025
Lin	2018	3	PRP	10.1097/MD.0000000000010477
Liu	2018	1	CSs, HA	10.1016/j.joms.2017.10.028
Machon	2018	2	AB	10.1007/s10006-017-0666-6
Moldez	2018	1	CSs, HA	10.11607/ofph.1783
Mustafa	2018	2	HD	10.1097/SCS.0000000000004480
Nagori	2018	1	HD	10.1111/joor.12698
Srinivas	2018	2	HA	10.5958/0974-360X.2018.00643.1
Sun	2018	3	HA	10.12659/MSM.908821
Vingender	2018	3	CSs, HA	10.1556/650.2018.31138
Yang	2018	2	HA	10.1016/j.joms.2018.04.031
Yapici-Yavuz	2018	2	CSs, HA, NSAID	10.4317/medoral.22237
Yoshida	2018	4	AB	10.1016/j.bjoms.2017.08.009
Al-Delayme	2017	4	PRP	10.1007/s12663-016-0911-9
Bouloux	2017	2	CSs, HA	10.1016/j.joms.2016.08.006
Castaño-Joaqui	2017	1	HA	10.1016/j.maxilo.2016.11.002
Cezairli	2017	4	HD	10.1089/acm.2017.0068
Gorrela	2017	2	HA	10.1007/s12663-016-0955-x
Guarda-Nardini	2017	4	HA	10.1080/08869634.2016.1232788
Gurung	2017	2	HA	10.4103/njms.NJMS_84_16
Iturriaga	2017	1	HA	10.1016/j.ijom.2017.01.014
Ozdamar	2017	2	HA	10.1111/joor.12467
Patel	2017	4	AB	10.4317/jced.53812
Peng	2017	4	HA	10.1016/j.ijom.2017.02.1219
Pihut	2017	3	HA, PRP	N/A
Refai	2017	4	HD	10.1016/j.bjoms.2016.12.002
Yang	2017	4	LPCGF	10.1097/MD.0000000000006302

N/A—not applicable; AB—autologous blood; BTX—botulinum toxin; CSs—corticosteroids; HA—hyaluronic acid; HD—hypertonic dextrose; I-PRF—injectable platelet-rich fibrin; LA—local anesthetics; LPCGF—liquid-phase concentrated growth factor; MSCs—mesenchymal stem cells; NSAID—non-steroidal anti-inflammatory drug; PRGF—plasma rich in growth factors; PRP—platelet-rich plasma.
